# Interband Photoconductivity of Metamorphic InAs/InGaAs Quantum Dots in the 1.3–1.55-μm Window

**DOI:** 10.1186/s11671-018-2524-3

**Published:** 2018-04-16

**Authors:** Sergii Golovynskyi, Oleksandr I. Datsenko, Luca Seravalli, Giovanna Trevisi, Paola Frigeri, Ivan S. Babichuk, Iuliia Golovynska, Junle Qu

**Affiliations:** 1 Key Laboratory of Optoelectronic Devices and Systems of Ministry of Education and Guangdong Province, College of Optoelectronic Engineering, Shenzhen University, Shenzhen, 518060 People’s Republic of China; 2 Institute of Semiconductor Physics, NAS of Ukraine, Kyiv, 03028 Ukraine; 3 Department of Physics, Taras Shevchenko National University of Kyiv, Kyiv, 01601 Ukraine; 4 Institute of Materials for Electronics and Magnetism, CNR-IMEM, I-43124 Parma, Italy

**Keywords:** Nanostructure, Quantum dot, Metamorphic, InAs/InGaAs, Photoconductivity, Photoluminescence, Photocurrent

## Abstract

Photoelectric properties of the metamorphic InAs/In_*x*_Ga_1 − *x*_As quantum dot (QD) nanostructures were studied at room temperature, employing photoconductivity (PC) and photoluminescence spectroscopies, electrical measurements, and theoretical modeling. Four samples with different stoichiometry of In_*x*_Ga_1 − *x*_As cladding layer have been grown: indium content *x* was 0.15, 0.24, 0.28, and 0.31. InAs/In_0.15_Ga_0.85_As QD structure was found to be photosensitive in the telecom range at 1.3 μm. As *x* increases, a redshift was observed for all the samples, the structure with *x* = 0.31 was found to be sensitive near 1.55 μm, i.e., at the third telecommunication window. Simultaneously, only a slight decrease in the QD PC was recorded for increasing *x*, thus confirming a good photoresponse comparable with the one of In_0.15_Ga_0.75_As structures and of GaAs-based QD nanostructures. Also, the PC reduction correlate with the similar reduction of photoluminescence intensity. By simulating theoretically the quantum energy system and carrier localization in QDs, we gained insight into the PC mechanism and were able to suggest reasons for the photocurrent reduction, by associating them with peculiar behavior of defects in such a type of structures. All this implies that metamorphic QDs with a high *x* are valid structures for optoelectronic infrared light-sensitive devices.

## Background

Metamorphic InAs/In_*x*_Ga_1 − *x*_As QD nanostructures have attracted much interest in the last decade owing to many benefits [1–7]. Their most attractive feature is that, by growing the QDs on an InGaAs metamorphic buffer (MB), one can achieve a significant reduction of the transition energy between the QD levels [8] with respect to conventional In(Ga)As/GaAs QD structures. This occurs due to the decrease of InAs QD bandgap as a result of the lattice mismatch reduction between InAs QDs and InGaAs buffer and, hence, the strain in QDs [9–11]. So, the application of a MB as a confining material allows to shift the emission wavelength value deeper into the infrared (IR) range, in particular, into the telecommunication windows at 1.3 and 1.55 μm, while maintaining a high efficiency [4, 12, 13]. Furthermore, metamorphic QDs have shown interesting properties such as (i) a high QD density [14], (ii) the possibility to widely tune QD and wetting layer (WL) levels [10, 15], and (iii) good performances of active elements in light-emitting devices [16]. However, the recent investigations of deep levels in metamorphic QDs showed that, despite InAs/In_0.15_Ga_0.85_As QD structures having a total defect density close to the QD layer comparable to that of InGaAs/GaAs pseudomorphic QDs, metamorphic structures with higher *x* demonstrated higher defect densities [17, 18].

Metamorphic InAs QD structures have found successful applications in the design and fabrication of IR photonic and light-sensitive devices, such as lasers [19, 20], single-photon sources [3, 7, 21, 22], and solar cells [23–25]. In(Ga)As QD photodetectors based on interband and intersubband transitions are currently actively investigated for enhanced detection from near-IR to longwave-IR ranges due to their response to the irradiation at normal incidence [26–30]. For instance, the intersubband transitions of electrons between quantum-confined levels and continuum states can be engineered by embedding InAs QDs in InGaAs layers [29–32], as this design allows to tune the detection peak wavelength, to control the response by an externally applied bias and to reduce the dark current [33, 34]. To date, there are no papers about the implementation of metamorphic QD structures in photodetectors.

The key role for the development of this area is the preservation of a high emission efficiency and photosensitivity of metamorphic QD structures that need to be at least comparable with those of conventional InAs/GaAs QD structures [1, 5, 35]. A lot of studies were carried out in the fundamental and application fields to develop structure design [6, 14, 21], to improve photoelectric properties [5, 13], and to control/reduce strain-related defects in the heterostructures [4, 36, 37].

Hence, InAs/In_*x*_Ga_1 − *x*_As metamorphic QD nanostructures are interesting nanostructures, which allow to have emission or photoresponsivity in the 1.3- and 1.55-μm IR ranges [1–7]. Furthermore, it was reported by us earlier that vertical InAs/In_0.15_Ga_0.75_As QD structures can maintain photosensitivity comparable to the GaAs-based ones [5]. However, such metamorphic structures are seldom studied in photoelectric measurements with a lateral geometry, where the photocurrent proceeds through in-plane transport of carriers across channels between two top contacts. Commonly, the QD layers along with the associated WL form these conductivity channels in the lateral geometry-designed GaAs-based structures [38]. Owing to this peculiar type of conductivity, QD photodetectors with the lateral transport are believed to have potential for a high photoresponsivity [39, 40]. An in-depth study of metamorphic InAs/InGaAs QD nanostructures in the lateral configuration can provide a fundamental knowledge about the photoconductivity (PC) mechanism and efficiency of the in-plain carrier transport. In our recent paper devoted to the defects in metamorphic QD structures [17], we reported lateral PC measurements at low temperatures, considering only the IR spectra edges originating from defects. However, we believe that a proper characterization and fundamental investigation of the structure at room temperature can give precious insights for further improvements of novel light-sensitive devices as near-IR photodetectors, linear arrays, and camera matrixes, by implementing metamorphic QDs.

In the present work, we studied in-plane photoelectric properties of the metamorphic InAs/In_*x*_Ga_1 − *x*_As QD nanostructures grown by molecular beam epitaxy with different In composition *x*, employing PC and photoluminescence (PL) spectroscopies, lateral electrical measurements, and modeling calculations. In particular, we focused on the observation of a possible redshift of the QD layer photoresponse toward the IR beyond 1.3 μm while preserving photosensitivity alike for In_0.15_Ga_0.85_As and GaAs QD light-sensitive structures. A high photosensitivity in the near-IR wavelength range at room temperature is an indication that these nanostructures can be useful not only for devices based on interband transitions but also for intersubband photodetectors working beyond 10 μm.

## Methods

### Sample Preparation and Description

**Fig. 1:**
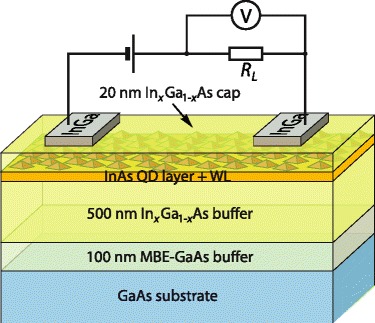
Color online. Scheme of the metamorphic InAs/In_*x*_Ga_1 − *x*_As QD structures and their connection for the photoelectric measurements

The studied structures schematically shown in Fig. 1 were grown by molecular beam epitaxy. Firstly, a semi-insulating (100) GaAs substrate was covered by a 100-nm thick GaAs buffer at 600 °C, followed by the deposition of an undoped InGaAs MB 500 nm in thickness at 490 °C. Then, after the prior growth interruption of 210 s to cool down the substrate, 3.0 MLs (monolayers) of InAs were grown at 460 °C. Finally, these self-assembled QDs were covered by 20 nm of undoped In_*x*_Ga_1 − *x*_As with the same MB stoichiometry. Four samples with different stoichiometry of In_*x*_Ga_1 − *x*_As cladding layer have been fabricated: In content *x* was 0.15, 0.24, 0.28, and 0.31.

### Theoretical Modeling

For metamorphic structure designing as well as understanding of the energy profile, the calculations of the quantum energy system composed by In(Ga)As QDs, undoped MB, and cap layer were carried out by using the Tibercad software [41] that we demonstrated to be adequate to simulate the optical properties of semiconductor low-dimensional nanostructures [2, 15, 42].

We consider an InAs QD with truncated conical shape and sizes taken from experimental atomic force microscopy data [14]; we include the presence of InAs WL, which parameters depend on the In_*x*_Ga_1 − *x*_As metamorphic layer properties [15].

First, strain calculations for the structure are made, by calculating the strain tensor components of the QD, induced by the mismatch *f*_QD_ between the QD and MB, defined as

1 $$ {f}_{\mathrm{QD}}=\left[{a}_{\mathrm{InAs}}\hbox{--} {a}_{\mathrm{MB}}(x)\right]/{a}_{\mathrm{MB}}(x) $$

where *a*_MB_(*x*) is the lattice parameter of In_*x*_Ga_1 − *x*_As MB and *a*_InAs_ is the lattice parameter of InAs. Then, band profiles for QDs and embedding layers depend on the deformation potentials of the relevant materials (InAs for QDs and WLs and relaxed InGaAs for MB).

Finally, the Schrödinger equation

2 $$ \boldsymbol{H}\psi = E\psi $$

is solved in the envelope function approximation by a single-band, effective-mass approach for electrons and 6 bands k•p approach for holes, where the 3D Hamiltonian is

3 $$ \widehat{H}=-\frac{\upeta^2}{2}{\nabla}_{\mathbf{r}}\left(\frac{1}{m\left(E,\mathbf{r}\right)}\right){\nabla}_{\mathbf{r}}+V\left(\mathbf{r}\right), $$

with *V*(**r**) being the 3D potential.

Such an approximation is considered satisfying when carrying on QD ground state calculation [2]. Ground levels for electrons and heavy holes are thus obtained, alongside their probability densities. Photoluminescence emission energies were derived by taking the energy difference between confined levels for electrons and heavy holes, reduced by 20 meV to take into consideration excitonic effects.

A more detailed description of model calculations can be found in Ref. [2].

### Photoelectric Characterization

**Fig. 2:**
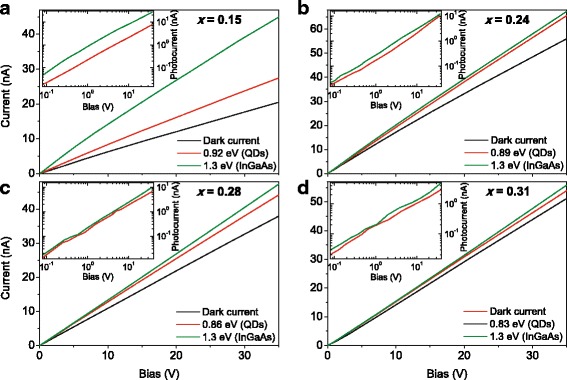
Color online. *I*–*V* characteristics of the InAs/In_*x*_Ga_1 − *x*_As structures with *x* = 0.15 (**a**), 0.24 (**b**), 0.28 (**c**), and 0.31 (**d**) for the dark (black) and under an illumination of 350 μW/cm^2^ (color) at energies of PL spectrum peak (QD excitation) and 1.3 eV (effective absorption in InGaAs). Insets: photocurrent dependences on bias voltage

For the lateral photoelectric measurements, two InGa eutectic surface contacts were deposited over 5 × 2 mm pieces of the structures. Measured linear *I*–*V* characteristics given in Fig. 2 have confirmed the contact ohmicity. The current flowing through the samples was measured by a Siglent SDM3055 multimeter, using a standard dc technique [43, 44] as a voltage drop across a series load resistance *R*_*L*_ of 1 MΩ, which was much less than the sample resistance. Photocurrent was excited by a 250-W halogen lamp light dispersed with a prism monochromer, and PC spectra were recorded in the range from 0.6 to 1.6 eV [44–46]. The spectra were normalized to the excitation quanta number of the light source. PL spectra were obtained using a 532-nm laser as an excitation source with a power density of 5 W/cm^2^. All the measurements were carried out at room temperature (300 K).

## Results and Discussion

**Fig. 3:**
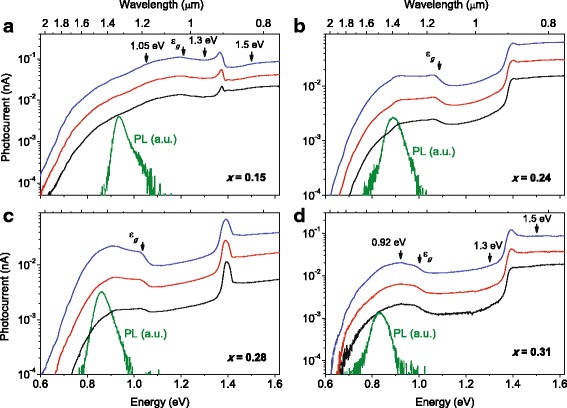
Color online. PC spectra of the metamorphic InAs/In_*x*_Ga_1 − *x*_As structures at room temperature and a bias of 11 V for *x* = 0.15 (**a**), 0.24 (**b**), 0.28 (**c**), and 0.31 (**d**). The excitation intensities for the black, red, and blue curves at 1.3 eV correspond to 88, 350, and 1400 μW/cm^2^, respectively. PL spectra in arbitrary units are given for the energy positioning of QD ground state transitions. The vertical arrows mark the InGaAs bandgaps (ε_*g*_) calculated following Paul et al. [48] and spectral positons, where the PC dependencies on excitation intensity were measured (given in Fig. 5)

**Fig. 4:**
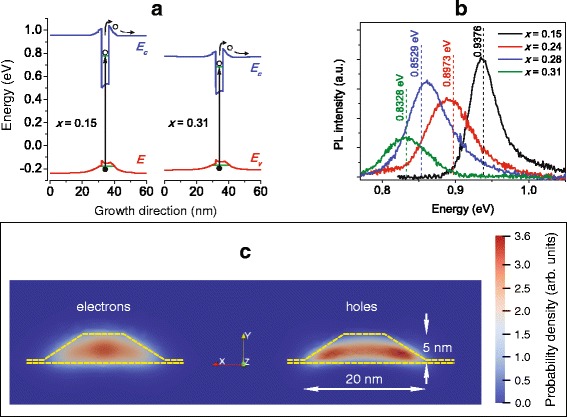
Color online. Modeling calculations for the metamorphic InAs/In_*x*_Ga_1 − *x*_As QD structures: **a** band profiles in the structures with different *x* along the growth axis; **b** the real QD PL bands and their calculated peak positions (dashed verticals); and **c** probability densities of the confined electrons and holes for the InAs/In_0.15_Ga_0.85_As QD. All the calculations of modeled structures were carried out for 300 K

PC spectra of the studied metamorphic InAs/In_*x*_Ga_1 − *x*_As QD structures at room temperature are given in Fig. 3 together with the PL bands, which show the optical transitions between QD ground states. The relative intensities and positions of the PL bands are also shown in Fig. 4b. Features due to the QDs, InGaAs confining layers, and GaAs bottom layers are observed on the PC curves. The photocurrent signal at the energies below the PL band onsets could be related to the structure defects detected earlier [17].

The investigated metamorphic InAs/In_0.15_Ga_0.85_As QD structure was found to be photosensitive in the telecom range at 0.95 eV (1.3 μm) (Fig. 3a). As *x* increased, a redshift was observed for all the samples: the structure with *x* = 0.31 was found to be sensitive near 0.8 eV (1.55 μm) (Fig. 3d), i.e., at the third telecom window [47]. The shift is related to the reduction of the lattice mismatch between the materials of InAs QD and In_*x*_Ga_1 − *x*_As buffer with an increase in *x* and, hence, a decrease in the strain in QDs. This leads to a narrowing of the InAs QD bandgap and, in turn, to the redshift of the PL band as well as the photoresponse onset toward IR [1–6, 19, 35].

Simultaneously, only a slight decrease in the QD photocurrent signal was recorded, thus confirming the preservation of a good photoresponsivity, comparable with that of the In_0.15_Ga_0.75_As sample. As we discussed recently [5], metamorphic QD structures with *x* = 0.15 show a photoresponse very similar to those of pseudomorphic InAs/GaAs QD nanostructures. Also, the PC reduction correlate with the PL one as it can be seen in Fig. 3.

Such an effect for our samples turned out to be most notable in Fig. 2, where the *I*–*V* dependences at the dark and under illumination at different characteristic spectral points on bias voltage are shown, together with the photocurrent dependences in the insets. Like in Fig. 3, the photocurrent value implies just the photoinduced part of current obtained from the total current under illumination by subtracting the dark current value. These spectral points are the PL band maximums and 1.3 eV, where an effective band-to-band absorption in InGaAs MB occurs. As well as for the dark *I*–*V* characteristics, these dependencies are linear-like within the experimental error.

The best photoresponse was measured in the structure with the minimal In content in the confining layers. It also had the lowest dark current. The photocurrent value at the applied excitation level (350 μW/cm^2^) in the InAs/In_0.15_Ga_0.85_As structure was two to three times above the dark current when MB was pumped. The photoresponse at QD excitation was comparable to the dark current; however, it should be considered that our structures had only one QD layer. Fabrication of the multilayered QD structures surely would lead to a significant increase in the IR photoresponse. Other structures with higher *x* revealed lower photocurrent signals; the detected magnitudes at both spectral points were approximately an order lower than the dark current values in a wide range of the applied voltage. The lowest photoresponse was found for the InAs/In_0.31_Ga_0.69_As structure with the maximal MB In content.

Most probably, this photoresponsivity decrease is related to an increase in the MB defect density with *x*, which was determined earlier for these structures, employing deep level thermally stimulated current spectroscopy [17], that correlated well with structural analysis of such nanostructures [1]. We have reported that the InAs/In_0.15_Ga_0.85_As QD structure had a total defect density close to the QD layer comparable to InGaAs/GaAs ones, whereas other structures with higher In contents demonstrated higher densities of defects like the known GaAs-related point defect complexes EL2, EL6, EL7, EL9, and EL10 near the QD layer and three levels attributed to extended defects propagating through the buffer.

In regard to the spectrum shape (Fig. 3), above the QD excitation, light absorption and, hence, the carrier generation occur mainly in the MB at energies above the InGaAs confining layer bandgap ε_*g*_, which values for different *x* were estimated by an empiric formula [48]. However, it is noteworthy that an increase in photon energy above ε_*g*_ leads to a slight decrease of the photoresponse. Naturally, this confirms that metamorphic QDs, despite being effective recombination centers [1, 2, 12, 22], are more efficient contributors to photocurrent than MB [5, 6, 23].

To understand the PC mechanism of this peculiarity, one should look at Fig. 4a, where we show the calculated QD band profiles along the growth direction for our samples. Calculations are validated by the result of quantum energy levels for electrons and holes: the expected PL emission energies are in agreement with the PL QD ground state transition measured experimentally (Fig. 4b). In Fig. 4c, we show the simulated probability densities for confined electrons and holes, obtained by the carrier wavefunctions calculated with the Tibercad modelization, that indicate a higher level of localization for heavy holes in comparison with electrons.

In order to contribute to the photocurrent signal, electron-hole pairs generated by QD interband absorption have to escape from QDs by thermal emission. In a previous study [49], it was established that in metamorphic QDs electrons and heavy holes escape simultaneously from QDs as correlated pairs. Moreover, it was also demonstrated that the activation energy for such process corresponds to the sum of the activation energies for the two particles [50].

While studying thermal quenching of PL emission from metamorphic QDs [10, 51], we proved that such activation energies are equal to the sum of the energy distance from the WL levels and QD states and go from 250 meV for *x* = 0.15 down to 150 meV for *x* = 0.31. As widely discussed in Ref. [51], these values cause a strong quenching of the PL emission at room temperature via the thermal escape of confined carriers.

On such basis, we can infer that carriers excited in QDs can thermally escape to WL and MB: there, electrons and heavy holes are separated by the band bending in the QD vicinity (Fig. 4a), which promotes the hole trapping back to QDs and, while being a barrier for electrons, thereby effectively suppresses their radiative recombination. As a consequence, heavy holes are concentrated at the QD periphery (Fig. 4c), whereas electrons are free to move along the potential well of WL and MB contributing to the conductivity. It is worth noting that in Ref. [49], it is discussed that, although correlated during the escape process, carriers cannot be considered as excitons at room temperature; henceforth, they can be easily separated by the band bending in the vicinity of QDs.

Otherwise, when exciting the MB, non-equilibrium holes are generated in the confining layers and do recombine with electrons. It should be mentioned here that the WL is known to be a conductivity channel for nanostructures based on GaAs [52] and, in our lateral structures designed with surface contacts, there is no heterojunction, so carriers are efficiently collected near the surface plane.

In Fig. 3, the fall of PC signal just above ε_*g*_ turned into the rise at higher energies, e.g., above 1.3 or 1.1 eV for sample with *x* of 0.15 or 0.31, respectively. This was conceivably caused by the optical absorption closer to the surface and QD layer, thus involving shallower traps. As established for these structures by thermally stimulated current spectroscopy and deep level transient spectroscopy [17, 18], the deeper electron traps are located mainly in the InGaAs MB layer, whereas the shallower ones are concentrated near the surface (in relation to these samples, near the QD layer). The electrons trapped into the shallower traps can more easily escape back to the conduction band at room temperature. Thus, free electrons near the QD layer are more mobile than those excited deeper in the MB and, hence, give a higher contribution to the charge transfer. Furthermore, the electrons, being generated near the surface, can freely transfer to the WL conductivity channel.

A similar drop of photocurrent following an increase above GaAs bandgap (near 1.4 eV) was observed. This effect might be due to the carrier generation close to the InGaAs/GaAs interface, which is known to have a higher density of defect states being traps and recombination centers.

**Fig. 5:**
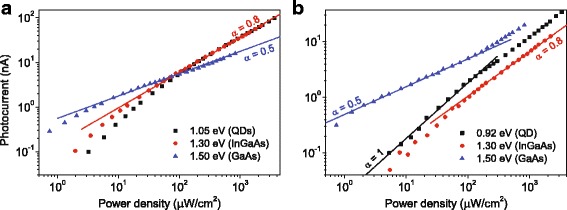
Color online. Photocurrent vs excitation intensity for the InAs/In_*x*_Ga_1 − *x*_As structures with **a** *x* = 0.15 and **b** 0.31. The lines are the fitting by functions *f*(*x*) ~ *x*^α^

The relative contribution of different optical transitions to the structure photoresponse varied with pumping intensity. This is better observed in Fig. 5, which shows photocurrent values as a function of the excitation intensity at different characteristic spectral points: the onset of the PL band (resonant excitation of the QD ensemble) or efficient band-to-band absorption in InGaAs (1.3 eV) and GaAs (1.5 eV).

The structures with different In contents in the confining layers demonstrated similar dependencies at equivalent spectral ranges. So, the band-to-band excitation in GaAs (1.5 eV) shows a quadratic dependence at most of intensity values. This is typical for the band-to-band recombination of non-equilibrium charge carriers, for instance when they are highly predominant under the equilibrium carriers [53]: this is expectable in our undoped structures. The dependencies in the case of excitation in the QD and InGaAs confining layers are very similar to each other but different from those for GaAs. They are linear at low excitation intensities and become sublinear at higher intensities. This behavior obviously points out to the carrier recombination involving Shockley-Read centers: the linear dependence becomes sublinear one, as some of the centers are saturated at higher carrier generation rates [54]. These results of intensity-dependent measurements distinctly indicate an efficient generation of main charge carriers at a relatively low recombination rate in QD embedding layers and a much higher density of recombination centers in GaAs layers. For example, during the QD excitation in similar characterizations, InGaAs/GaAs QD photosensitive structures showed a dependence from intensity as *PC*(*I*) ~ *I*^0.25^, which occurred due to a high rate of the non-radiative recombination though defect levels along with QD radiative recombination [40, 55]. However, it is worth to notice that the InGaAs/GaAs structure was multilayered having seven QD layers.

From these measurements and their interpretation, some indications for the use of metamorphic QDs for IR detection can be highlighted: (i) when using *x* > 0.15, advanced designs allowing to control strain-related defects should be used, similar to what was done for the development of metamorphic QDs [19, 20, 37]; (ii) multilayer stacks of QDs (with a minimum of 10 layers) are needed to obtain a QD PC above the dark current [27, 56]; and (iii) as a higher confinements of heavy holes is beneficial for the photocurrent obtained when exciting QDs, advanced designs with higher-gap barriers for heavy holes could be considered [51, 57]. Hence, these findings can be very useful for the design of metamorphic QDs aiming at IR detection and the development of metamorphic QD photodetectors.

## Conclusions

Photoelectric properties of the metamorphic InAs/In_*x*_Ga_1 − *x*_As QD nanostructures were studied at room temperature, employing PC and PL spectroscopies, electrical measurements, and theoretical model simulations. The studied metamorphic InAs/In_*x*_Ga_1 − *x*_As QD nanostructures were found to be photosensitive in the telecommunication windows at 1.3 (*x* = 0.15) and 1.55 μm (*x* = 0.31). However, the QD PC as well as the PL efficiencies of the structures with higher In contents in MB were estimated to be lower and, nevertheless, comparable to that of the InAs/In_0.15_Ga_0.85_As structure, which has sensitivity similar to InGaAs/GaAs QD structures. This photoresponsivity reduction is related to an increase in the MB defect density with *x*. Also, thanks to modeling calculations, we provided insights into the PC mechanism in the investigated type of QD structures. All this implies that metamorphic QDs with a high *x* are valid structures for optoelectronic IR light-sensitive devices, provided that some points of concern are addressed by optimization of the design of the nanostructure.

## Abbreviations

ε_*g*_: Bandgap of the InGaAs confining layer
*E*_c_ and *E*_v_: Energy of conductivity and valence bands
IR: Infrared
MB: Metamorphic buffer
ML: Monolayer
PC: Photoconductivity
PL: Photoluminescence
QD: Quantum dot
*R*_L_: The load resistance
WL: Wetting layer

## References

[1] Seravalli L, Frigeri P, Nasi L, Trevisi G, Bocchi C (2010). Metamorphic quantum dots: quite different nanostructures. J Appl Phys.

[2] Gioannini M, Cedola AP, Di Santo N, Bertazzi F, Cappelluti F (2013). Simulation of quantum dot solar cells including carrier intersubband dynamics and transport. IEEE J Photovoltaics.

[3] Munoz-Matutano G, Barrera D, Fernandez-Pousa CR, Chulia-Jordan R, Seravalli L, Trevisi G (2016). All-optical fiber Hanbury Brown & Twiss interferometer to study 1300 nm single photon emission of a metamorphic InAs quantum dot. Sci Rep.

[4] Semenova ES, Zhukov AE, Mikhrin SS, Egorov AY, Odnoblyudov VA, Vasil’ev AP (2004). Metamorphic growth for application in long-wavelength (1.3-1.55 μm) lasers and MODFET-type structures on GaAs substrates. Nanotechnology.

[5] Golovynskyi S, Seravalli L, Datsenko O, Trevisi G, Frigeri P, Gombia E (2017). Comparative study of photoelectric properties of metamorphic InAs/InGaAs and InAs/GaAs quantum dot structures. Nanoscale Res Lett.

[6] Golovynskyi S, Seravalli L, Datsenko O, Kozak O, Kondratenko SV, Trevisi G (2017). Bipolar effects in photovoltage of metamorphic InAs/InGaAs/GaAs quantum dot heterostructures: characterization and design solutions for light-sensitive devices. Nanoscale Res Lett.

[7] Paul M, Olbrich F, Höschele J, Schreier S, Kettler J, Portalupi SL (2017). Single-photon emission at 1.55 μm from MOVPE-grown InAs quantum dots on InGaAs/GaAs metamorphic buffers. Appl Phys Lett.

[8] Trevisi G, Seravalli L, Frigeri P, Prezioso M, Rimada JC, Gombia E (2009). The effects of quantum dot coverage in InAs/(In)GaAs nanostructures for long wavelength emission. Microelectron J.

[9] Seravalli L, Minelli M, Frigeri P, Allegri P, Avanzini V, Franchi S (2003). The effect of strain on tuning of light emission energy of InAs/InGaAs quantum dot nanostructures. Appl Phys Lett.

[10] Seravalli L, Minelli M, Frigeri P, Franchi S, Guizzetti G, Patrini M (2007). Quantum dot strain engineering of InAs/InGaAs nanostructures. J Appl Phys.

[11] Wang P, Chen QM, Wu XY, Cao CF, Wang SM, Gong Q (2016). Detailed study of the influence of InGaAs matrix on the strain reduction in the InAs dot-in-well structure. Nanoscale Res Lett.

[12] Seravalli L, Frigeri P, Trevisi G, Franchi S (2008). 1.59 μm room temperature emission from metamorphic InAs/InGaAs quantum dots grown on GaAs substrates. Appl Phys Lett.

[13] Golovynskyi SL, Seravalli L, Trevisi G, Frigeri P, Gombia E, Dacenko OI (2015). Photoelectric properties of the metamorphic InAs/InGaAs quantum dot structure at room temperature. J Appl Phys.

[14] Seravalli L, Trevisi G, Frigeri P (2012). 2D-3D growth transition in metamorphic InAs/InGaAs quantum dots. CrystEngComm.

[15] Seravalli L, Trevisi G, Frigeri P (2013). Calculation of metamorphic two-dimensional quantum energy system: application to wetting layer states in InAs/InGaAs metamorphic quantum dot nanostructures. J Appl Phys.

[16] Mi Z, Wu C, Yang J, Bhattacharya P (2008). Molecular beam epitaxial growth and characteristics of 1.52 μm metamorphic InAs quantum dot lasers on GaAs. J Vac Sci Technol.

[17] Golovynskyi S, Datsenko O, Seravalli L, Kozak O, Trevisi G, Frigeri P (2017). Deep levels in metamorphic InAs/InGaAs quantum dot structures with different composition of the embedding layers. Semicond Sci Technol.

[18] Rimada JC, Prezioso M, Nasi L, Gombia E, Mosca R, Trevisi G (2009). Electrical and structural characterization of InAs/InGaAs quantum dot structures on GaAs. Mater Sci Eng B-Adv.

[19] Mi Z, Bhattacharya P (2008). Pseudomorphic and metamorphic quantum dot heterostructures for long-wavelength lasers on GaAs and Si (invited paper). IEEE J Sel Top Quant.

[20] Karachinsky LY, Kettler T, Novikov II, Shernyakov YM, Gordeev NY, Maximov MV (2006). Metamorphic 1.5 μm-range quantum dot lasers on a GaAs substrate. Semicond Sci Technol.

[21] Seravalli L, Trevisi G, Frigeri P (2012). Design and growth of metamorphic InAs/InGaAs quantum dots for single photon emission in the telecom window. CrystEngComm.

[22] Seravalli L, Trevisi G, Frigeri P, Rivas D, Munoz-Matutano G, Suarez I (2011). Single quantum dot emission at telecom wavelengths from metamorphic InAs/InGaAs nanostructures grown on GaAs substrates. Appl Phys Lett.

[23] Azeza B, Alouane MHH, Ilahi B, Patriarche G, Sfaxi L, Fouzri A (2015). Towards InAs/InGaAs/GaAs quantum dot solar cells directly grown on Si substrate. Materials.

[24] Rouis W, Haggui M, Rekaya S, Sfaxi L, M’ghaieth R, Maaref H (2016). Local photocurrent mapping of InAs/InGaAs/GaPts intermediate-band solar cells using scanning near-field optical microscopy. Sol Energ Mat Sol C.

[25] Han IS, Kim JS, Kim JO, Noh SK, Lee SJ (2016). Fabrication and characterization of InAs/InGaAs sub-monolayer quantum dot solar cell with dot-in-a-well structure. Curr Appl Phys.

[26] Wu J, Chen SM, Seeds A, Liu HY (2015). Quantum dot optoelectronic devices: lasers, photodetectors and solar cells. J Phys D Appl Phys.

[27] Passmore BS, Jiang W, Manasreh MO, Kunets VP, Lytvyn PM, Salamo GJ (2008). Room temperature near-infrared photoresponse based on interband transitions in In_0.35_Ga_0.65_As multiple quantum dot photodetector. IEEE Electron Device Lett.

[28] Kondratenko SV, Iliash SA, Vakulenko OV, Mazur YI, Benamara M, Marega E (2017). Photoconductivity relaxation mechanisms of InGaAs/GaAs quantum dot chain structures. Nanoscale Res Lett.

[29] Shao J, Vandervelde TE, Barve A, Stintz A, Krishna S (2012). Increased normal incidence photocurrent in quantum dot infrared photodetectors. Appl Phys Lett.

[30] Vaillancourt J, Stintz A, Meisner MJ, Lu XJ (2009). Low-bias, high-temperature operation of an InAs-InGaAs quantum-dot infrared photodetector with peak-detection wavelength of 11.7 μm. Infrared Phys Technol.

[31] Lu X, Meisner MJ, Vaillancourt J, Li J, Liu W, Qian X, Goodhue WD (2007). Modulation-doped InAs-InGaAs quantum dot longwave infrared photodetector with high quantum efficiency. Electron Lett.

[32] Lu XJ, Vaillancourt J, Meisner MJ, Stintz A (2007). Long wave infrared InAs-InGaAs quantum-dot infrared photodetector with high operating temperature over 170K. J Phys D Appl Phys.

[33] Lin W-H, Chao K-P, Tseng C-C, Mai S-C, Lin S-Y, Wu M-C (2009). The influence of In composition on InGaAs-capped InAs/GaAs quantum-dot infrared photodetectors. J Appl Phys.

[34] Nedzinskas R, Čechavičius B, Rimkus A, Pozingytė E, Kavaliauskas J, Valušis G (2015). Temperature-dependent modulated reflectance of InAs/InGaAs/GaAs quantum dots-in-a-well infrared photodetectors. J Appl Phys.

[35] Seravalli L, Frigeri P, Minelli M, Franchi S, Allegri P, Avanzini V (2006). Metamorphic self-assembled quantum dot nanostructures. Mat Sci Eng C-Bio S.

[36] Mi Z, Bhattacharya P, Yang J (2006). Growth and characteristics of ultralow threshold 1.45 μm metamorphic InAs tunnel injection quantum dot lasers on GaAs. Appl Phys Lett.

[37] Mazzucato S, Nardin D, Capizzi M, Polimeni A, Frova A, Seravalli L (2005). Defect passivation in strain engineered InAs/(InGa)As quantum dots. Mat Sci Eng C-Bio S.

[38] Kunets Vas P, Rebello Sousa Dias M, Rembert T, Ware ME, Mazur Yu I, Lopez-Richard V (2013). Electron transport in quantum dot chains: dimensionality effects and hopping conductance. J Appl Phys.

[39] Towe E, Pan D (2000). Semiconductor quantum-dot nanostructures: their application in a new class of infrared photodetectors. J Sel Top Quantum Electron.

[40] Golovynskyi SL, Dacenko OI, Kondratenko SV, Lavoryk SR, Mazur YI, Wang ZM (2016). Intensity-dependent nonlinearity of the lateral photoconductivity in InGaAs/GaAs dot-chain structures. J Appl Phys.

[41] Auf der Maur M, Penazzi G, Romano G, Sacconi F, Pecchia A, Di Carlo A (2011). The multiscale paradigm in electronic device simulation. IEEE Trans Electron Devices.

[42] Trevisi G, Seravalli L, Frigeri P (2016). Photoluminescence monitoring of oxide formation and surface state passivation on InAs quantum dots exposed to water vapor. Nano Res.

[43] Kondratenko SV, Vakulenko OV, Mazur YI, Dorogan VG, Marega E, Benamara M (2014). Deep level centers and their role in photoconductivity transients of InGaAs/GaAs quantum dot chains. J Appl Phys.

[44] Vakulenko OV, Golovynskyi SL, Kondratenko SV (2011). Effect of carrier capture by deep levels on lateral photoconductivity of InGaAs/GaAs quantum dot structures. J Appl Phys.

[45] Kondratenko SV, Golovinskiy SL, Vakulenko OV, Kozyrev YN, Rubezhanska MY, Vodyanitsky AI (2007). Photocurrent spectroscopy of indirect transitions in Ge/Si multilayer quantum dots at room temperature. Surf Sci.

[46] Valakh MY, Dzhagan VM, Yukhymchuk VO, Vakulenko OV, Kondratenko SV, Nikolenko AS (2007). Optical and photoelectrical properties of GeSi nanoislands. Semicond Sci Technol.

[47] Song H-Z, Hadi M, Zheng Y, Shen B, Zhang L, Ren Z (2017). InGaAsP/InP nanocavity for single-photon source at 1.55-μm telecommunication band. Nanoscale Res Lett.

[48] Paul S, Roy JB, Basu PK (1991). Empirical expressions for the alloy composition and temperature-dependence of the bandbap and intrinsic carrier density in Ga_x_In_1-x_As. J Appl Phys.

[49] Sanguinetti S, Colombo D, Guzzi M, Grilli E, Gurioli M, Seravalli L (2006). Carrier thermodynamics in InAs/In_x_Ga_1−x_As quantum dots. Phys Rev B.

[50] Le Ru EC, Fack J, Murray R (2003). Temperature and excitation density dependence of the photoluminescence from annealed InAs/GaAs quantum dots. Phys Rev B.

[51] Seravalli L, Trevisi G, Frigeri P, Franchi S, Geddo M, Guizzetti G (2009). The role of wetting layer states on the emission efficiency of InAs/InGaAs metamorphic quantum dot nanostructures. Nanotechnology.

[52] Danil’tsev VM, Drozdov MN, Moldavskaya LD, Shashkin VI, Germanenko AV, Min’kov GM (2004). Electron transport effects in the IR photoconductivity of InGaAs/GaAs structures with quantum dots. Tech Phys Lett.

[53] Sze SM, Ng KK (2006). Physics of semiconductor devices.

[54] Duboc CA (1955). Nonlinearity in photoconducting phosphors. Br J Appl Phys.

[55] Golovynskyi SL, Mazur YI, Wang ZM, Ware ME, Vakulenko OV, Tarasov GG (2014). Excitation intensity dependence of lateral photocurrent in InGaAs/GaAs dot-chain structures. Phys Lett A.

[56] Ezzedini M, Hidouri T, Alouane MHH, Sayari A, Shalaan E, Chauvin N (2017). Detecting spatially localized exciton in self-organized InAs/InGaAs quantum dot superlattices: a way to improve the photovoltaic efficiency. Nanoscale Res Lett.

[57] Seravalli L, Frigeri P, Allegri P, Avanzini V, Franchi S (2007). Metamorphic quantum dot nanostructures for long wavelength operation with enhanced emission efficiency. Mater Sci Eng C.

